# Characteristics Associated with Homeless Pregnant Women in Columbus, Ohio

**DOI:** 10.1007/s10995-021-03227-y

**Published:** 2021-10-06

**Authors:** Emma Ervin, Barbara Poppe, Amanda Onwuka, Hannah Keedy, Stephen Metraux, Leslie Jones, Megan Sandel, Kelly Kelleher

**Affiliations:** 1 The Abigail Wexner Research Institute at Nationwide Children’s Hospital, Columbus, OH USA; 2 Barbara Poppe and Associates, Columbus, OH USA; 3 Biden School of Public Policy & Administration, University of Delaware, Newark, DE USA; 4 Boston Medical Center, Boston, MA USA

**Keywords:** Homeless, Pregnancy, Housing, Maternal, Prenatal

## Abstract

**Introduction:**

The effects of homelessness on pregnant women are substantial. We aim to identify key characteristics of a group of women identified as homeless and pregnant in order to understand their history of housing, family composition, health, and demographics as a first step for future intervention.

**Methods:**

We present cross-sectional survey data on a sample of 100 women reporting homelessness and pregnancy in the prior year in Columbus, Ohio, identified through social service and housing not for profit agencies. Our analysis uses data collected from a survey of health behaviors, housing, employment status, and demographics. Continuous measures are described with means and standard deviations, and categorical variables are described with percentages.

**Results:**

The majority (81%) of the women identified as African American. Over 95% of the women were single, and 74 women reported a prior pregnancy. Almost half of the women reported being behind on rent at least one time in the last 6 months, and 43% indicated that they had lived in more than three places in the last year. Approximately 34% of the sample reported cigarette use during pregnancy, while 12% and 30% reported alcohol and illicit drug use, respectively.

**Discussion:**

Women who were pregnant and experiencing homelessness in our study reported a multitude of complex and severe problems ranging from high rates of substance use, longstanding housing insecurity and financial stress. Programs hoping to successfully support women will need to address a variety of service needs while recognizing the resilience of many women.

## Significance Statement

While much is known about the effects of homelessness on women, gaps remain regarding specific characteristics, circumstances and the frequency of these challenges. Identifying possible areas of intervention for women who are homeless and pregnant is key in decreasing rates on infant mortality, preterm birth and low birth weight. The focus of interventions should be to improve housing and health.

## Introduction

The affordable housing crisis in the United States (U.S.) led to declines in affordable housing and increased rates of homelessness. In the past decade, there has been a 17% increase in the number of households spending more than 50% of their income on rent (up from 5.7 million in 2007 to 6.7 million in 2017) (Henry et al., 2018). On any given night in January of 2018 in the U.S., about 552,380 individuals were homeless and nearly 30% identified as women or girls. In addition, homelessness in women increased by 3% in 2018 from the previous year according to the most recent report from the U.S. Department of Housing and Urban Development. (Henry et al., 2018). This increase is especially concerning for females of child-bearing age and pregnant women due to the impacts these factors can have on reproductive and maternal health, birth outcomes, and child health (Clark et al., 2019; Sandel et al., 2015; Stein et al., 2000).

Reasons for homelessness can often go unidentified and unaddressed. However, some known causes of homelessness in women are domestic violence, evictions or other family problems (Tischler et al., 2007). One study suggests that 80% of homeless mothers with children had previously experienced domestic violence (Aratani, 2009). Another study described patterns of pregnancy, itself, being a cause of homelessness for many adolescent women as the new child would contribute to crowding in the home or was not welcome.

The myriad of stressors associated with homelessness include, but are not limited to, inadequate nutrition, lack of sleep and limited access to medical care. These stressors may have important effects on mothers and children. Exposure to air pollution and poor nutrition increase the likelihood of preterm birth and low birth weight (Lorch & Enlow, 2016; Mehra et al., 2017; Collins et al., 2004). The presence of lead in the home, rodent and pest infestations, crowding, temperature concerns and bed-sharing have also been found to be associated with poor birth outcomes (Andrews et al., 1994; Burris & Hacker, 2017).

Although health stressors and risks are well described, intervention trials and the multiple areas of need for women experiencing homelessness and pregnancy are almost absent from the literature. Many studies intervene with pregnant women who have numerous risk factors, but these interventions are predominantly ‘home’ visiting programs. For women experiencing homelessness, too, a small number of studies have examined housing first and case management models. Unfortunately, these studies have numerous methodological problems and provide limited evidence of effectiveness (Krahn et al., 2018). Moreover, factors associated with keeping women housed like longstanding medical problems, utility and eviction factors are not often assessed.

The effects of homelessness on pregnant women are substantial. We aim to identify key characteristics and circumstances of a group of women identified as homeless and pregnant in order to further develop interventions for future multi-site, randomized trials. We present cross-sectional data on a sample of 100 women reporting homelessness and pregnancy in the prior year in Columbus, Ohio, a large Midwestern city, to examine their history of housing insecurity, family composition, and health problems.

## Methods

Our cross-sectional analysis uses survey data collected from the screening for a housing program for pregnant women, a randomized control trial. Surveys were administered to capture key information about extremely low-income, Medicaid-eligible, pregnant women who lived in a large Midwestern county in Ohio. Eligibility information was collected from the women during the intake survey. The remainder of the survey was administered after enrollment and the intake survey was complete. The surveys focused on health behaviors, housing affordability, rental stability, food security, employment status and demographics.

To be a participant, women had to be 18 or older, housing insecure, in their first or second trimester at time of enrollment and have a household income of less than 30% of the area median income (AMI). In order to be considered housing unstable or homeless, the women must have demonstrated one of the following in the last 12 months: multiple prior moves, a history of eviction or at risk of eviction, overcrowded or doubled up, or a severe housing problem. The definition of housing unstable is one that varies by source. We consulted with Children’s Health Watch who has written extensively on this topic. The criteria chosen involved literature searches and affiliated partner input.” Participants also must have been enrolled in CareSource (an Ohio Medicaid company), owed less than $1000 for utility arrearages, been willing to complete a credit check and criminal background check, and consented to data sharing among the various partners. Criminal background checks were examined for arson and drug manufacturing charges which were exclusionary criteria. Consents and surveys were available in Spanish and English versions.

One hundred women were verbally consented and enrolled to complete research surveys. Surveys were performed over the phone or via email, and survey results were entered into the REDCap database (Harris et al., 2009). Colleagues at the University of Delaware analyzed intake data obtained by program staff from each program enrollee (see Tables 1 and 3 where n = 100). Continuous measures are described with means and standard deviations, and categorical variables are described with percentages. Calculations were completed using SPSS version 26.

**Table 1:** Characteristics

	Total (n = 91)	
Race/ethnicity		
Non-hispanic Black/African American	74	81%
Non-hispanic white	9	10%
Other^a^	8	9%
Age in years (Mean, SD)	25.5	4.6
Gestational age in weeks (Mean, SD)	18.2	5.5
Primary language		
English	91	100%
Education attained		
Some high school	25	27%
High school graduate or GED	47	52%
Some college, vocational, or technical school	19	21%

	Total (n = 100)	
Adults in household		
One	83	83%
Two	16	16%
Missing	1	1%
Prior criminal history	44	44%
Income per month		
$0–$500	55	55%
$501–$1000 +	45	45%
Credit score		
Above 580 (“low” or “average”)	8	8%
Below 580 (“bad” or “poor”)	38	38%
No score (insufficient information)	54	54%

^a^Hispanic Black/African American or multiracial

## Results

Ninety-one of the 100 women completed their full survey between August 2018 and February 2019. After enrollment into the study, three women asked to be withdrawn, one woman was unable to complete the survey due to a language barrier, and five women did not complete their survey. However, all 100 women had consented to data sharing during their enrollment; therefore, intake analyses include a sample of 100.

### Demographics

Table 1 presents demographic characteristics of the pregnant women who were experiencing highly unstable housing situations. The majority (81%) of these women identified as African-American. The average age of women was 25.5 years (SD 4.6). Average gestational age at enrollment was 18 weeks (SD 5.5), because women were required to be in their first or second trimester. All the women surveyed spoke English as their primary language. Over 95% of the women were single, however, 16 of the women reported having one other adult in the household. The majority (52%) of the women have either finished high school or received a GED and 27% lacked a high school diploma. Approximately 44% of the sample indicated a prior criminal record. Just over half of the total group (55%) reported an income of zero to five-hundred dollars during the previous month at time of enrollment and the majority (92%) of the women had a credit score below 580 (considered poor by crediting reporting agencies). At the time of the full survey, 59% of women reported that they were unemployed.

### Circumstances

Table 2 presents pregnancy history data. A total of 74 women in the program (81%) reported a prior pregnancy. Of these 74 women, forty-three had lost a child through still birth or miscarriage, thirteen had a baby born with a low birth weight, and eighteen had a baby born premature.

**Table 2:** Pregnancy history

	Total (n = 91)	
Prior pregnancy		
Yes	74	81%
No	17	19%

	Total (n = 74)	
Prior miscarriage or stillbirth	43	58%
Prior low birth weight	13	18%
Prior premature baby	18	24%

Table 3 contains data regarding the housing history during the last 12 months (unless otherwise noted) of the enrolled women. At the time of enrollment in the program, eleven women were either living in a car, on the streets, in an abandoned building, in a homeless shelter, or in a residential drug and alcohol treatment program. An additional 65 women indicated that they were staying with friends or family; and fifteen women indicated they were living in a house or apartment that they rent. Almost half of the women reported being behind on rent at least one time in the last 6 months and 43% of the sample indicated that they had lived in more than three places in the last year. The majority of the women, approximately 53%, experienced a period of homelessness that lasted for a few months or longer. Forty-four women (48%) expressed concerns about paying rent on time or concerns for eviction, and 21 women (23%) expressed a desire to live in a safer neighborhood. Thirty-six women (40%) indicated concerns of a risk of domestic violence in their household. Among women with concerns about housing conditions, problems with utilities, plumbing, infestations and mold/mildew were reported. Over one third of the women experienced at least one eviction while 62% and 54% had electric and gas arrears, respectively.

**Table 3:** Housing history

	Total (n = 91)	
Current living arrangement		
Living in a house/apartment that I rent	15	16%
Staying with friends/family	65	71%
Other	11	12%
Late on rent in last 6 months	43	47%
Three or more places lived in last year	39	43%
Time spent homeless		
A few months or less	29	32%
Most to all of the year	28	31%
My family was never homeless	34	37%
Voiced concerns		
Paying rent	35	38%
Poor housing conditions	19	21%
Eviction/want to avoid an eviction	30	33%
Needing to move into a shelter	28	31%
Risk of domestic violence	36	40%
Wanting to be closer to work, school, or family	56	62%
Wanting a safer neighborhood	21	23%
A change in your family	65	71%
Having your own place to stay	44	48%
Getting kicked out	8	9%
Housing quality concerns		
Utilities shut off/not working	10	11%
Plumbing problems	9	10%
Current infestations	13	14%
Presence of mold	6	7%

	Total (n = 100)	
Number of times evicted		
None	59	59%
One	17	17%
Two or more	24	24%
Electric bill arrears		
Yes	62	62%
Missing	1	1%
Gas bill arrears		
Yes	54	54%
Missing	1	1%

### Health Behaviors and Characteristics

Table 4 includes relevant health behaviors and characteristics of the women who were enrolled. Common pregnancy complications in this sample during their current pregnancy include vaginal bleeding (n = 17), preeclampsia (n = 7), infections such as rubella, chicken pox, urinary tract infection, or sexually transmitted infection (n = 27), and diabetes during pregnancy (n = 8). Approximately 34% of the sample reported cigarette use during pregnancy while 12% reported alcohol use and 30% reported illicit drug use. Nearly two-thirds of the women reported being in good, fair, or poor health with only twenty-eight women reporting excellent or very good health. The majority of the enrolled women reported little to no exercise per week while twelve women reported working out five or more times per week. Nearly 45% of the women reported a previous diagnosis of depression, however, just 19% of women reported taking anti-depressants. Anemia was also prevalent among this sample with 43% of women reporting previous diagnosis. Over 90% of the women reported use of prenatal vitamins or supplements at time of enrollment into the program.

**Table 4:** Health behaviors

	Total (n = 91)	
Inadequate weight gain during this pregnancy	11	12%
Pregnancy complications		
Vaginal bleeding	17	19%
Preeclampsia	7	8%
Infections	27	30%
Diabetes during pregnancy	8	9%
None	39	43%
Cigarette use	31	34%
Alcohol use	11	12%
Drug use		
One or more	27	30%
None	64	70%
General health		
Excellent/very good	28	31%
Good/ fair/poor	63	69%
Exercise frequency		
None	48	53%
1–2 times per week	24	26%
3 or more times per week	19	20%
Prior health conditions		
Hypertension	6	7%
Diabetes	10	11%
Anemia	39	42%
Depression	40	44%
Obesity	12	13%
Medications		
Anti-depressants	17	19%
Prenatal vitamins	85	93%

## Discussion

Prior studies outlined the serious threats to maternal/child health when pregnant women experience homelessness and even housing insecurity. This study adds to that prior literature in underscoring the significant financial distress and multiple comorbidities experienced by such women in one city.

The women in our study described serious concerns with paying rent, eviction, and housing quality. Nearly half of the women (46%) reported an income of zero dollars during the previous month, and extensive electric and gas bill arrears. Most of the women (92%) had either a low or no credit score. All of these factors have been found to impact housing security and homelessness (Beal & Redlener, 1995; Shinn & Weitzman, 1990). Moreover, prior arrears in utilities and a complete lack of income make personal level initiatives meaningless in obtaining housing. Systemic housing solutions are needed to overcome such barriers.

Low income pregnant women face an inhospitable housing market in urban areas, nationally. Most low-income families spend more than 50% of their income on housing in this metropolitan area, which is often of compromised quality (The Columbus and Franklin County Affordable Housing Challenge: Needs, Resources, and Funding Models, 2017). Additionally, rodent and pest infestations, crowding, and poor temperature control are common experiences among low-income families, and these conditions are also risk factors for infant mortality (Andrews et al., 1994; Burris & Hacker, 2017; Chu et al., 2016). Furthermore, low-income housing is often located in neighborhoods with poor air quality, compromised safety and racial isolation, which are community factors associated with increased risk of adverse birth outcomes (Anthopolos et al., 2014; Collins et al., 2004; Lorch & Enlow, 2016; Mehra et al., 2017; Mendez et al., 2014). Approximately half of the women enrolled in the study experienced homelessness that lasted a few months or longer during the last 12 months.

Our study also highlights the very high-risk maternity histories for women experiencing homelessness during pregnancy. 4% experienced a still birth, 24% had a prior preterm delivery, and 18% had a prior low birth weight baby. These high-risk maternity histories are higher than the national averages reported by the CDC in 2017. Despite the myriad of challenges faced by participants around housing and health, over 90% of the women reported using prenatal vitamins, well over the average of 77% found in a nationally representative sample of pregnant women (Branum et al., 2013).

Less novel, but consistent with prior research, our study found high rates of domestic violence (40%) and illicit drug use (28%) during pregnancy for women experiencing homelessness (Domestic Violence & Homelessness: Statistics, 2016). A study by Pavao suggests that women who experienced interpersonal violence in the last year had nearly four times the odds of housing insecurity or homelessness than women who did not experience interpersonal violence (Pavao et al., 2007).

While this study is informative, it also has limitations. First, all participants were from an urban county in Columbus, Ohio. A sample from a rural area could have drastically different characteristics and needs. Our study also prioritized women from CelebrateOne neighborhoods (i.e., neighborhoods with the highest rates of infant mortality in Franklin County). These geographical areas see higher unemployment, lower graduation rates, increased homelessness, lack of access to nutritious food, higher instances of crime and limited access to health coverage. These factors further limit comparison to populations in other residential settings. Finally, all survey participants were recruited from a single Medicaid managed care organization (CareSource). We lack data from women who were not able to sustain Medicaid coverage, chose a different plan, or did not meet the income requirements for state-funded insurance.

## Conclusions for Practice

These surveys were conducted in the process of developing a new, pilot intervention program for women who were pregnant and experiencing homelessness. The findings, if generalizable to other sites, suggest that the profound financial and maternity risk involved requires more than a usual case management and health care coordination if our focus is on maternal child health. Added components should include significant additional financial resources for offsetting utility arrears, much longer-term rent support than many housing first programs, and higher than expected security deposits. In addition, intensive prenatal care that addresses prior preterm births and other morbidities must be tightly integrated.
